# Preparation and Characterization of Body-Temperature-Responsive Thermoset Shape Memory Polyurethane for Medical Applications

**DOI:** 10.3390/polym15153193

**Published:** 2023-07-27

**Authors:** Xiaoqing Yang, Zhipeng Han, Chengqi Jia, Tianjiao Wang, Xiaomeng Wang, Fanqi Hu, Hui Zhang, Jun Zhao, Xuesong Zhang

**Affiliations:** 1 Department of Orthopedics, The First Affiliated Hospital of Jinan University, Guangzhou 510630, China; yangxiaoqing301@126.com; 2 Department of Orthopaedics, The Fourth Medical Centre, Chinese PLA General Hospital, Beijing 100048, China; danielchia@163.com (C.J.); hufanqi301@163.com (F.H.); 3 CAS Key Laboratory of Nanosystem and Hierarchical Fabrication, CAS Center for Excellence in Nanoscience, National Center for Nanoscience and Technology, Beijing 100190, China; hanzp1995@163.com (Z.H.); wtj869357204@163.com (T.W.); bradywang01@163.com (X.W.); zhangh@nanoctr.cn (H.Z.); 4 Tianjin Key Laboratory of Molecular Optoelectronic Sciences, Department of Chemistry, School of Science, Tianjin University & Collaborative Innovation Center of Chemical Science and Engineering (Tianjin), Tianjin 300072, China; 5 Medical School of Chinese PLA, Beijing 100853, China; 6 Department of Orthopedics, Beijing Jishuitan Hospital, Capital Medical University, Beijing 100035, China; 7 Research Institute of Aerospace Special Materials and Processing Technology, Beijing 100074, China; 8 AVIC Manufacturing Technology Institute, Beijing 101300, China; 9 University of Chinese Academy of Sciences, Beijing 100049, China

**Keywords:** shape memory polymer, polyurethane, thermoset, body temperature responsive, medical applications

## Abstract

Shape memory polymers (SMPs) are currently one of the most attractive smart materials expected to replace traditional shape memory alloys and ceramics (SMAs and SMCs, respectively) in some fields because of their unique properties of high deformability, low density, easy processing, and low cost. As one of the most popular SMPs, shape memory polyurethane (SMPU) has received extensive attention in the fields of biomedicine and smart textiles due to its biocompatibility and adjustable thermal transition temperature. However, its laborious synthesis, limitation to thermal response, poor conductivity, and low modulus limit its wider application. In this work, biocompatible poly(ε-caprolactone) diol (PCL-2OH) is used as the soft segment, isophorone diisocyanate (IPDI) is used as the hard segment, and glycerol (GL) is used as the crosslinking agent to prepare thermoset SMPU with a thermal transition temperature close to body temperature for convenient medical applications. The effects of different soft-chain molecular weights and crosslinking densities on the SMPU’s properties are studied. It is determined that the SMPU has the best comprehensive performance when the molar ratio of IPDI:PCL-2OH:GL is 2:1.5:0.33, which can trigger shape memory recovery at body temperature and maintain 450% recoverable strain. Such materials are excellent candidates for medical devices and can make great contributions to human health.

## 1. Introduction

Shape memory polymers (SMPs) have broad potential applications in aerospace [1], medicine and health [2,3,4], industrial control [5,6,7], surface engineering [8,9], self-healing [10], 3D printing [11,12,13,14,15,16], and other fields [17,18] because they can keep a temporary shape at room temperature and restore to their original shape when stimulated by the outside world [19,20,21,22,23,24]. SMPs are expected to replace traditional shape memory alloys and ceramics (SMAs and SMCs, respectively) in some fields and have become the most intensively studied shape memory materials [25,26,27,28,29,30,31,32,33,34]. Compared with other popular SMPs, such as polyolefin [35] and epoxy resin [36,37,38,39], shape memory polyurethane (SMPU) has wider applications in fields such as biomedicine and smart textiles because of its high degree of deformability, easy processing, adjustable thermal transition temperature, and biocompatibility [40]. However, its arduous chemical synthesis, limitation to thermal response, poor electrical and thermal conductivity, and low mechanical modulus still need to be improved to make it suitable for real-world applications.

As one component of many PU materials, poly(*ε*-caprolactone) (PCL) is a linear, semi-crystalline polymer formed by the ring-opening polymerization of *ε*-caprolactone monomer [41]. The five -CH_2_ groups and one polar -COO- group in the repeating unit endow it with good flexibility and processability and excellent biocompatibility and degradability, making it suitable for applications in the biomedical field. With increased molecular weight, the mechanical properties of PCL are improved, but its transition temperature (*T*_trans_) also increases. Excessive *T*_trans_ makes it difficult to be used in body-temperature-responsive biomedical devices. Furthermore, linear PCL does not have a highly elastic state and melts into a liquid at high temperatures. In order to achieve a pronounced shape memory effect, peroxide chemical reaction or high-energy ray radiation is usually required to obtain crosslinking points in such materials.

When used alone as a shape memory material, PCL usually faces the problem of poor mechanical properties. To overcome this problem, in our previous work, by selecting PCL diols (PCL-2OH) and PCL triols (PCL-3OH) as the prepolymers, a one-step method was employed to prepare a two-way shape memory polyurethane (2W-SMPU) with high reversible strain and high shape stability [42]. The PCL-3OH with a low melting temperature (*T*_m_) was used as an actuator to fix the temporary shape, and the PCL-2OH with a higher *T*_m_ was used as a skeleton to maintain the permanent shape. Thus, PCL-3OH was directed to crystallize along the PCL-2OH skeleton, and a chemical, crosslinked network structure was generated between PCL-2OH and PCL-3OH.

Based on our previous work involving the use of copolymers with PCL-2OH [42] and considering future applications of the prepared SMPU in biomedicine and other near-body-temperature fields [43,44], we abandon the traditional toxic dibutyltin dilaurate catalyst in the synthesis process and choose a more environmentally friendly and non-toxic organic bismuth catalyst. PCL-2OH is used as the soft segment, and isophorone diisocyanate (IPDI) and glycerol (GL) are used as the hard segment and crosslinking agent, respectively. By selecting the appropriate molecular weight and appropriately adjusting the raw material ratio, a simple one-step method is used to prepare a large deformation-recoverable SMPU with a *T*_trans_ of about 37 °C and a good shape memory effect. In this work, we not only accurately control the response temperature of SMPU to near body temperature by optimizing the raw materials and reaction conditions but also significantly improve its mechanical properties and shape memory effect, enabling large-scale and rapid preparation, which is conducive to the widespread application of this type of material in the biomedical field.

## 2. Experimental Part

### 2.1. Materials

**Figure 1:**
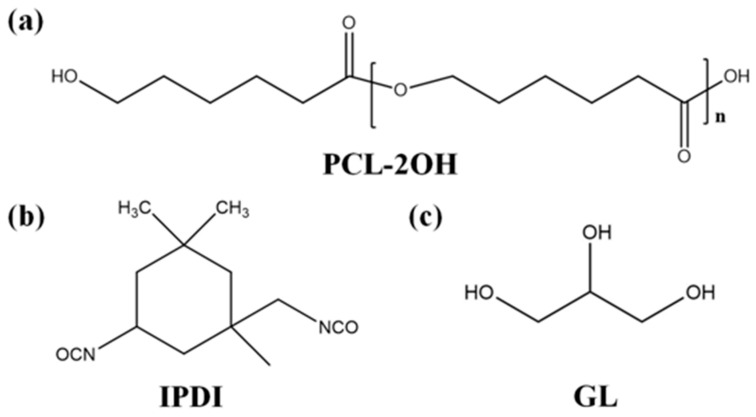
Chemical structure formulae of (**a**) PCL-2OH, (**b**) IPDI, and (**c**) GL.

PCL-2OH, purchased from Shenzhen Yisheng New Materials Co. Ltd., China, is a milky white solid at room temperature and needs to be melted in a 90 °C oven before use. Detailed information on PCL-2OH with various molecular weights is shown in Table 1. IPDI of analytical purity, purchased from Shanghai McLean Co., China, is a liquid at room temperature and has a molecular weight of 220 g mol^−1^. GL of analytical purity, provided by Beijing Chemical Factory, China, is a liquid at room temperature and has a molecular weight of 92 g mol^−1^. After removing GL’s moisture in an oven at 80 °C, some molecular sieve was added to the GL. The organic bismuth catalyst (DY-20) with a bismuth content of 20 ± 0.5 wt%, purchased from Shanghai Deyin Chemical Co., China, has the main component of bismuth new caprate and is a light yellow transparent liquid at room temperature with a density of 1.15 g·cm^−3^. *N*,*N*-dimethylformamide (DMF) of analytical purity was supplied by Beijing Chemical Factory, China. Figure 1 shows the chemical structure formulae of PCL-2OH, IPDI, and GL.

### 2.2. Test Instruments

Table 2 lists the main instruments and equipment used in this work.

### 2.3. Preparation of Samples

A predetermined amount of PCL-2OH was weighed and placed in a three-necked flask. Then, it was dried in a vacuum oven at 120 °C for 3 h. IPDI and GL in a predetermined ratio were weighed and placed in a beaker; 5 mL of DMF was then added to the flask and beaker, respectively, to form solutions for later use. The three-necked flask was taken out and electromagnetically stirred at 250 rpm for 30 min in a nitrogen environment and then naturally cooled to about 70 °C. Then, the above IPDI and GL solution was slowly added and stirred evenly for 5 min. In the flask, the solution gradually turned white from transparent. After further stirring for 2 min, the solution was poured into a beaker and then put in a vacuum oven at 25 °C for the defoaming treatment for 1 min. Afterwards, the solution was slowly poured into a preheated mold at 50 °C and placed in an oven at 50 °C for curing. The curing procedure was 50 °C for 3 h, 70 °C for 3 h, and then 100 °C for 10 h, based on the previous experimental experience [42]. A too-high heating temperature in the early stage might accelerate the volatilization of DMF and result in the generation of a large number of bubbles in the sample.

### 2.4. Material Characterization

The Fourier transform infrared spectroscopy (FTIR) was used to conduct the infrared spectroscopy tests on the synthesized SMPU to characterize the molecular structure and functional groups of the materials. The material was tested in the air as a film without further processing. The tests were in total reflection mode; the scanning range was 4000–450 cm^−1^; the resolution was 4 cm^−1^; and the scanning was repeated 8 times.

Scanning electron microscopy (SEM) was used to observe the fracture morphology of the SMPU. The accelerating voltage was 5–15 kV. The sample was fractured at low temperature in liquid nitrogen, then attached to a *T*-shaped sample stage with conductive adhesive, and sputtered with gold for 50 s before the observation on the SEM.

Differential scanning calorimetry (DSC) was used to test the *T*_trans_ and melting enthalpy change (Δ*H*_m_) in SMPU, where the position of the maximum melting peak was taken as the *T*_trans_. The tests were in a standard mode, and the nitrogen flow rate was 50 mL·min^−1^. First, the thermal history of the sample was removed by increasing the temperature from room temperature to 130 °C at a heating rate of 10 °C·min^−1^, and then the sample was cooled down to −70 °C at a cooling rate of 10 °C·min^−1^. Subsequently, the sample was heated again from room temperature to 130 °C at a heating rate of 10 °C·min^−1^. The last heating curve was taken to determine the *T*_trans_ and Δ*H*_m_.

Thermogravimetric analysis (TGA) was used to analyze the thermal stability of the samples. The tests were performed by increasing the temperature from room temperature to 800 °C at a heating rate of 10 °C·min^−1^. The nitrogen flow rate was 60 mL·min^−1^, and the air flow rate was 40 mL·min^−1^.

Dynamic mechanical thermal analysis (DMTA) was used to test the quasi-static stress–strain, storage modulus, and shape memory properties of the samples. The samples were cut into 15.0 × 5.0 × 1.0 mm^3^ rectangular splines. For the quasi-static stress–strain tests, the strain rate mode was chosen, and the temperature was raised to 55 °C at a heating rate of 10 °C min^−1^, and then the temperature was kept constant for 5 min to ensure the crystals melted in the materials and to make the overall temperature reach 55 °C, followed by stretching to 100% strain at a strain rate of 50% strain min^−1^. For the storage modulus tests, the multi-frequency-strain mode was chosen, and the frequency was set to 1 Hz. The temperature was increased from 0 °C to 85 °C at a heating rate of 5 °C min^−1^. For the tensile shape memory tests, the control force mode was chosen, and the temperature was first increased from room temperature to 55 °C at a heating rate of 10 °C·min^−1^, followed by keeping the temperature constant for 5 min. Then, stress was applied to the sample at a rate of 0.5 MPa·min^−1^, and the next step was performed when the deformation reached about 50–70% strain. The temperature was decreased to 0 °C at a cooling rate of 10 °C min^−1^, followed by keeping the temperature constant for 10 min, and then the stress was completely unloaded at a rate of 0.5 MPa min^−1^. Finally, the temperature was raised to 55 °C at a heating rate of 10 °C·min^−1^, and the temperature was kept constant for 20 min to ensure the material reached the maximum recovery degree. The shape memory performance was characterized by the shape fixation ratio *R*_f_ and the shape recovery ratio *R*_r_, which were calculated as follows:

(1) $$R_{f}=\frac{\varepsilon_{fix}}{\varepsilon_{load}}\times 100\%$$

(2) $$R_{r}=\frac{\varepsilon_{fix}-\varepsilon_{rec}}{\varepsilon_{fix}}\times 100\%$$

where *ε*_load_ was the maximum strain achieved by the material under the high-temperature load stress, *ε*_fix_ was the fixed strain of the material after the stress was unloaded at the low temperature, and *ε*_rec_ was the strain of the material after the final heating and shape recovery.

X-ray diffractometry (XRD) was used to test the crystallization properties of the samples. The diffraction angle range was from 5° to 90° at a scanning speed of 10° min^−1^.

The gel fraction was measured to see the degree of crosslinking of materials by their weight losses. A small part of the sample was cut and weighed to obtain the weight of *w*_i_. The sample was soaked in a bottle of DMF solvent for 12 h and then taken out, dried, and weighed. Such a procedure was repeated several times until the difference from the last weighing was less than 1 wt%, and the last weight was *w*_f_. So the gel fraction (*f*_g_) was calculated as follows [42]:

(3) $$f_{g}=\frac{w_{f}}{w_{i}}\times 100\%$$

## 3. Results and Discussion

### 3.1. Influence of Different Molecular Weights

The thermally responsive SMPU is formed by the reaction of polyisocyanates containing isocyanate groups (-N=C=O) with polyether or polyester polyols containing active hydrogen. The urethane groups generated in the reaction can interact with the urethane groups in other segments. It produces hydrogen bonding and has higher *T*_trans_, so it is mostly used as a hard segment, and polyether or polyester polyol is used as a soft segment. The thermal, mechanical, and shape memory properties of SMPU are closely related to the type and molecular weight of the soft segment. Based on the equivalent molar ratio of IPDI:PCL-2OH:GL = 2:1.5:0.33, in which the molar ratio of -NCO:-OH is exactly 1:1, we selected PCL-2OH with molecular weights of 500, 1000, 2000, and 3000 g·mol^−1^, respectively, to prepare the SMPU and explore the effect of soft chain length on the material properties. The prepared samples are marked as SMPU-05, SMPU-10, SMPU-20, and SMPU-30, respectively.

**Figure 2:**
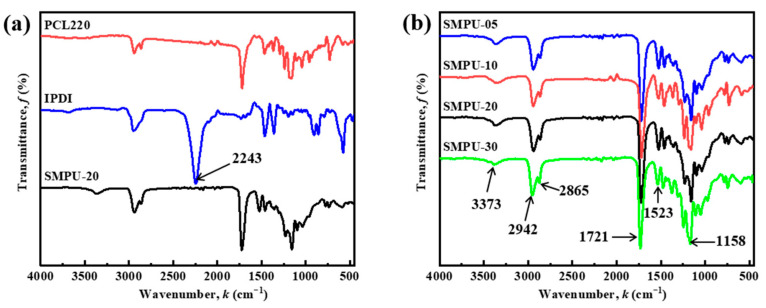
FTIR spectra of different samples: (**a**) comparison of raw materials and products, and (**b**) SMPU synthesized from PCL-2OH with different molecular weights.

The chemical structure of the as-synthesized SMPU was analyzed by using FTIR. As shown in Figure 2, the absorption peak of the free =N-H group is around 3460 cm^−1^, and the absorption peak of the hydrogen-bonded =N-H group is around 3310 cm^−1^. When there are hydrogen bonds between molecules, the stretching vibration peak of the =N-H group moves to the lower wavenumber region, so the absorption peak of =N-H group is found at 3373 cm^−1^, which indicates that part of the =N-H group in the molecular chain is hydrogen-bonded. The =C=O group exists in the carbamate group and in the ester group of PCL-2OH, and the absorption peak of =C=O in the free state is located at 1695 cm^−1^. The absorption peak of hydrogen bonding in the ordered phase is at 1635–1645 cm^−1^, so the absorption peak at 1721 cm^−1^ indicates that the molecular chain =C=O is not hydrogen-bonded. The peak at 1635–1645 cm^−1^ is the stretching vibration absorption peak of ≡C-H in SMPU, the peak at 1523 cm^−1^ is the deformation vibration absorption peak of =N-H, and the peak at 1158 cm^−1^ is the stretching vibration absorption peak of ≡C-O-C≡ vibration absorption peak. As shown in Figure 2a, the IPDI exhibits a characteristic peak belonging to –N=C=O at 2243 cm^−1^, which disappears without any residue in the four synthesized SMPU products, indicating that all the –N=C=O groups have reacted with the -OH groups in the PCL-2OH and GL. Therefore, it can be preliminarily determined that the synthesized SMPU is non-toxic, and the above results show that the polyurethane (PU) materials have been successfully synthesized by the one-step method.

**Figure 3:**
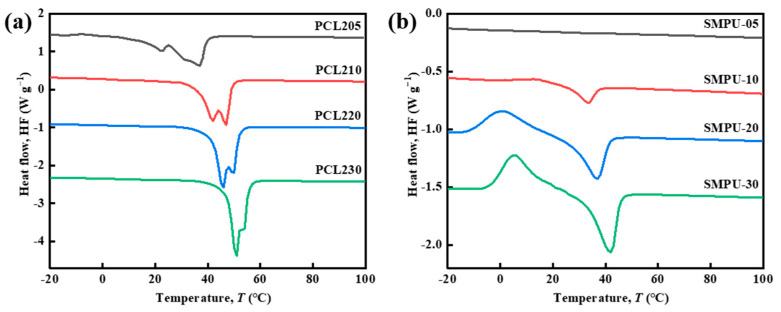
DSC curves of different samples: (**a**) PCL-2OH with different molecular weights, and (**b**) SMPU synthesized by PCL-2OH with different molecular weights. The endothermic is in the downward direction.

Figure 3 shows the DSC curves of PCL-2OH with different molecular weights and the synthesized SMPU, whose *T*_trans_ and Δ*H*_m_ are listed in Table 3. It can be seen that the higher the molecular weight of PCL-2OH, the higher the *T*_trans_, the higher the peak height, and the narrower the peak width, which means that the longer the molecular chain of PCL-2OH, the easier it is to crystallize. The enthalpy change can be used to evaluate the crystallization properties of the material. Different from the changing trend of *T*_trans_, the Δ*H*_m_ of PCL-2OH is 70–80 J g^−1^, and there is no significant difference. The melting enthalpy of perfect PCL crystals is about 140 J g^−1^, indicating that PCL-2OH is a semi-crystalline polymer with crystallinity of 52.7–56.1%. Here, based on the Δ*H*_m_ of the corresponding PCL-2OH, the crystallization melting enthalpy change value is calculated for comparison. The crystallinities of SMPU-05 to SMPU-30 are 0%, 5.8%, 20.6%, and 26.1%, respectively.

Compared with the PCL-2OH, the *T*_trans_ and Δ*H*_m_ of the synthesized SMPU decrease significantly to different degrees, and the melting peak even completely disappears for SMPU-05. The *T*_trans_ of SMPU-20 appears at 36.9 °C, which is very close to the temperature of the human body. The appearance of the peak is related to the melting of soft segment crystals. With the increase of the molecular weight of PCL-2OH, the *T*_trans_ increases, and the Δ*H*_m_ also increases significantly. The cold crystallization peak appears for the SMPU-20 and SMPU-30 and moves to the higher temperature region with the increase of *T*_trans_. The appearance of the cold crystallization peak is mainly due to the formation of a supramolecular network structure in the synthesized SMPU after the GL crosslinking, and the motion of the PCL soft segment is bound. The mobility of the chain segment is enhanced, the crystallization continues, and the exothermic phenomenon occurs during the heating, resulting in the emergence of a cold crystallization peak. The Δ*H*_m_ of the SMPU is smaller than that of PCL-2OH, indicating that part of PCL-2OH in the material does not participate in the crystallization but reacts with IPDI to form the hard-segment urethane groups. The incomplete participation of PCL-2OH in the crystallization makes the prepared SMPU have lower *T*_trans_, but at the same time, sacrifices the crystallizability of PCL, which is manifested as lower Δ*H*_m_, and is not conducive to the expected shape memory effect.

**Figure 4:**
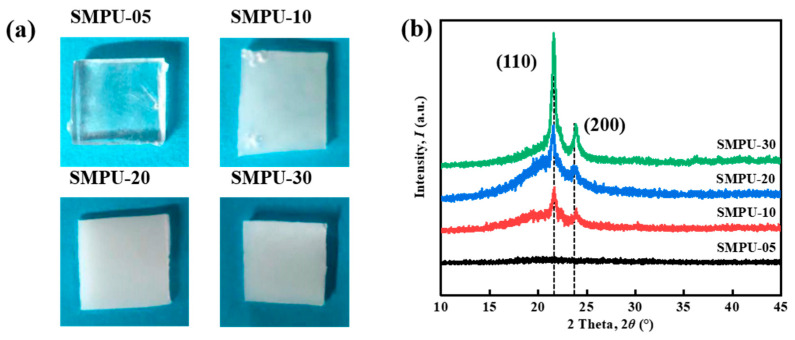
(**a**) Optical images and (**b**) XRD patterns of the SMPU with different soft chain molecular weights.

Figure 4a shows a series of optical pictures of the SMPU at room temperature. It can be seen that SMPU-05 is completely transparent. With the increase of the molecular weight of the soft chain used, the transparency of SMPU decreases significantly and finally appears as a milky white opaque solid. This is due to the existence of two phases in SMPU: one part is an amorphous phase, and the other part is a crystalline phase formed by a part of PCL-2OH soft chain crystallization. The refractive indices of the two are different, and light cannot be transmitted out after multiple refractions between the two phases, thus showing milky white. The milky white color in the material might also indicate the crystallization of soft chains in SMPU, forming a good two-phase separation, which is helpful for the generation of shape memory function.

In order to verify that the longer soft chain makes the polymer easier to crystallize, XRD characterization was used to observe the crystallization of the materials, and the results are shown in Figure 4b. In the XRD pattern, there are two sharp diffraction peaks at 21.2° and 23.5°, which belong to the (110) and (200) crystal planes, respectively. These reflection peaks are characteristic peaks of PCL orthorhombic unit cells. The crystallization peak area represents the crystallinity of the material, and the larger the peak area, the higher the crystallinity. In the spectrum, as the molecular weight of the soft segment chain increases, the two diffraction peaks of SMPU gradually increase, and the degree of crystallinity in SMPU-30 is much higher than that in SMPU-05. The XRD results are consistent with the DSC results, indicating that the crystallinity of SMPU is affected by the molecular weight of the soft segment, and the longer the soft segment chain, the higher the crystallinity.

**Figure 5:**
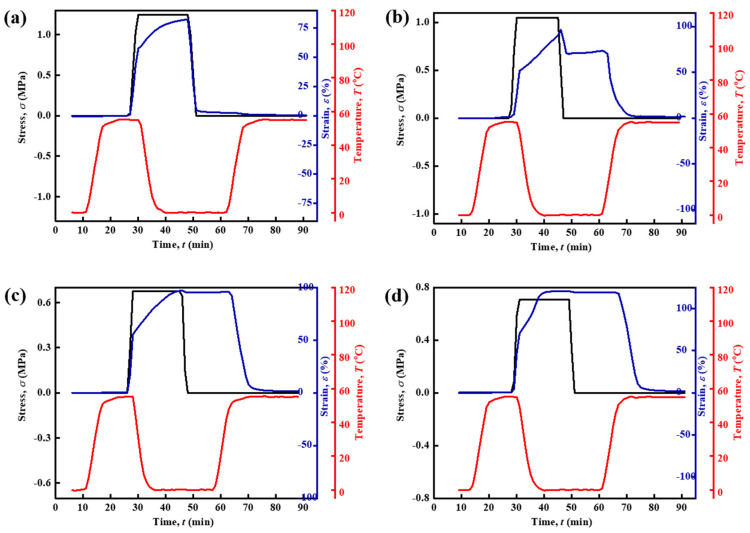
Shape memory curves of SMPU with different soft chain molecular weights: (**a**) SMPU-05, (**b**) SMPU-10, (**c**) SMPU-20, and (**d**) SMPU-30.

Furthermore, the shape memory function of the materials was tested. Figure 5 shows the shape memory process of the four SMPUs, including the maximum strain *ε*_load_ under load stress, the fixed strain *ε*_fix_ after stress removal, and the recovered strain *ε*_rec_ at high temperatures. These data and the statistical calculation results of the shape fixation ratio *R*_f_ and the shape recovery ratio *R*_r_ are listed in Table 4. From Figure 5, it can be seen that the shape of SMPU-05 recovers immediately after the stress is unloaded, and the *R*_f_ is only 3.1%. The reason for its extremely low shape-fixing function could be related to its short soft chain, which is difficult to crystallize. As the molecular weight of the soft chain increases, the *R*_f_ of the material increases rapidly, reaching 76.1% for the molecular weight of 1000 g mol^−1^ and obtaining almost perfect shape fixation (>95%) for the molecular weight of 2000 g mol^−1^. At the same time, it also has a perfect *R*_r_ (about 98.4%).

**Figure 6:**
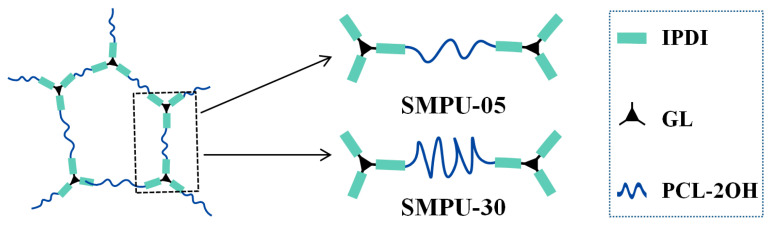
Difference between the soft segments of SMPU-05 and SMPU-30.

In summary, according to the above results of FTIR, DSC, XRD, and shape memory tests on the SMPU with different soft chain molecular weights, it can be seen that the shape memory performance of SMPU is closely related to the molecular weight of the soft chain that determines the crystallizability of the soft segment, thereby affecting the shape fixation ratio of the material. The soft chains with higher molecular weights are easier to crystallize and thus have a better shape fixation ratio, while those with lower molecular weights are more difficult to crystallize or do not crystallize at all, so they have a poorer shape fixation ratio. As shown in Figure 6, PCL-2OH reacts with IPDI to form the hard-segment carbamate groups and is crosslinked by GL. Although the two ends are fixed, the middle part of the chain segment with higher molecular weight can continue to move and agglomerate together to form crystals, so the longer the chain segment, the easier it is to crystallize. There are melting peaks and diffraction peaks appearing and gradually increasing, which also explains why the SMPU synthesized with PCL-2OH has a lower *T*_trans_, and both the *T*_trans_ and Δ*H*_m_ of SMPU increase with the increase in the molecular weight of the soft segment.

### 3.2. Influence of the Crosslinking Density

According to the shape memory test results, the SMPU-05 prepared by PCL205 exhibits pure elastic rubber behavior, which is completely unsuitable for the preparation of SMPU. In contrast, the SMPU-10 prepared by PCL210 has a good shape recovery ratio (*R*_r_ > 95%) but is lacking in the *R*_f_ (<80%). The SMPU-20 and SMPU-30 have excellent shape memory performance (*R*_f_ > 95%, *R*_r_ > 95%), indicating that PCL220 and PCL230 are both suitable for the preparation of excellent SMPU. During the shape memory testing, it was also noticed that as the molecular weight of the soft segment increases, the stress required to achieve the desired strain decreases, probably due to the higher crosslinking density in the SMPU with shorter soft segments per unit volume. As a result, under the same stress conditions, the strain achieved by SMPU-30 is larger than that of SPMU-20, which to some extent, indicates its worse mechanical properties. Secondly, the *T*_trans_ of SMPU-20 is 36.9 °C, which is closer to the human body temperature, while the *T*_trans_ of SMPU-30 is 42.3 °C, and the human body can already feel a little burning pain at this temperature. After careful consideration, PCL220 is chosen to prepare the SMPU for further exploration.

**Figure 7:**
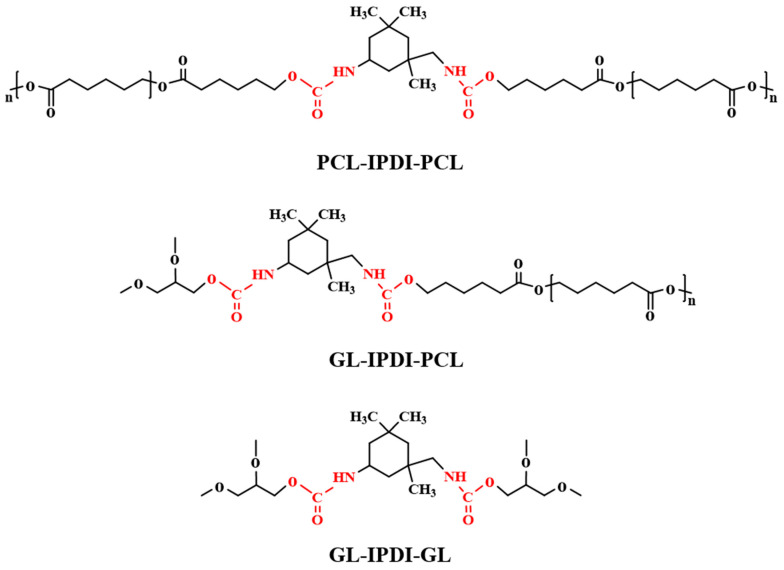
Three connection modes in the crosslinked network of SMPU. The formed amide groups are marked in red.

The raw materials for the one-step synthesis of SMPU are mainly IPDI, PCL-2OH, and GL. During the curing, three connection methods are mainly produced by the reaction to build the crosslinking network, namely PCL-IPDI-PCL type, PCL-IPDI-GL type, and GL-IPDI-GL type, as shown in Figure 7. In the case of ensuring that -NCO:-OH is 1:1, the crosslinking density is changed by changing the molar ratio of PCL-2OH to GL so as to realize the effect of crosslinking density on the thermal, crystalline, and shape memory properties of the materials. Therefore, four kinds of materials with PCL-2OH:GL molar ratios of 1.00:0.67, 1.25:0.50, 1.50:0.33, and 1.75:0.17 were prepared according to the molar ratio. The prepared samples are marked with GL content, respectively, as SMPU(0.67), SMPU(0.50), SMPU(0.33), and SMPU(0.17), respectively. The numbers in the brackets not only represent the molar ratio of GL but also reflect the crosslinking density.

**Figure 8:**
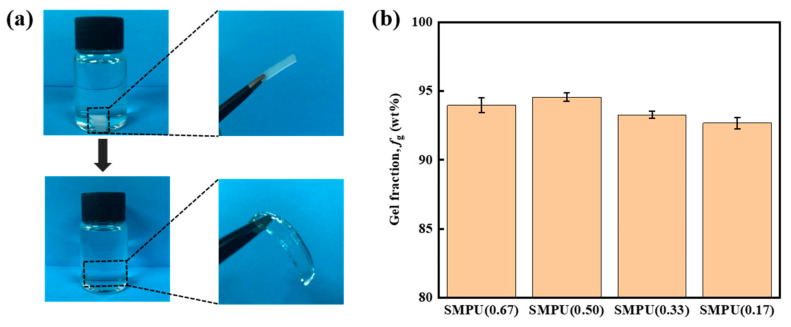
(**a**) Image contrast before and after the gel fraction test, and (**b**) the gel fraction test results of different SMPU samples.

The crosslinking degree test was carried out on the four SMPU materials. The SMPU in different compositions was put into the DMF solvent, and the volume of the materials expanded, as shown in Figure 8a. The pores of the crosslinking network became larger, and the uncrosslinked part of the materials was extracted into the DMF solvent. As shown in Figure 8b, the gel fraction of all the samples is above 90 wt%, indicating that most molecules are involved in the construction of the crosslinking network. On the overall trend, as the crosslinking density decreases, the proportion of PCL-2OH in the material increases, and the gel fraction shows a downward trend. It is speculated that there are two reasons. First, some IPDI or GL volatilizes during the curing, resulting in the unbalanced molar ratio of -NCO:-OH. Due to the steric hindrance effect, IPDI will first react with the GL of small molecules, leaving a small part of PCL-2OH that does not participate in the reaction. Second, as the proportion of PCL-2OH in the material increases, some PCL-2OH reacts with IPDI to form linear PU, which will not participate in the construction of the crosslinking network and is washed out in the gel fraction tests.

**Figure 9:**
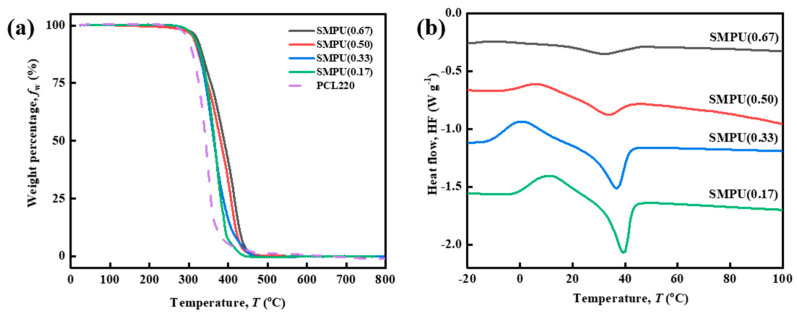
Thermal properties of SMPU with different crosslinking densities: (**a**) TGA curves and (**b**) DSC curves. For part b, the endothermic is in the downward direction.

In terms of thermal properties, DSC and TGA were used to test the *T*_trans_ and the thermal stability of the four materials, respectively. Figure 9a shows the TGA curves of the four materials, and it can be seen that compared with PCL-2OH, the thermal stability of the SMPU is improved. The weight loss of 5 wt% is defined as the initial decomposition temperature of the sample. At around 310 °C, all materials begin to decompose gradually, and the initial decomposition temperature is not significantly different. SMPU(0.17) and SMPU(0.33) enter the stage of rapid weight loss first, but there is little difference in the final decomposition temperature between them. SMPU(0.17) completes the decomposition first, and all the samples reach 95 wt% weight loss at about 430 °C. Overall, the higher the crosslinking density, the better the thermal stability of the materials, but the difference is not significant.

The *T*_trans_ of the four materials were tested by DSC. From Figure 9b, it can be seen that the melting peak of SMPU(0.67) is almost invisible, and the *T*_trans_ is about 32.3 °C (see Table 5). As the crosslinking density decreases, the proportion of PCL-2OH in the material increases, and the *T*_trans_ of the polymer gradually increases. For SMPU(0.17), the *T*_trans_ reaches about 39.7 °C. The Δ*H*_m_ also increases with the increase of the proportion of PCL-2OH in the material, showing the largest melting peak in SMPU(0.17), which is narrower and higher than all other peaks. DSC tests show that the lower the crosslinking density, the higher the *T*_trans_ of the polymer, and the narrower and higher the melting peak, which should be due to the more PCL soft chains in SMPU participating in the crystallization, and the formation of crystallization is beneficial to the shape fixing in the shape memory process.

**Figure 10:**
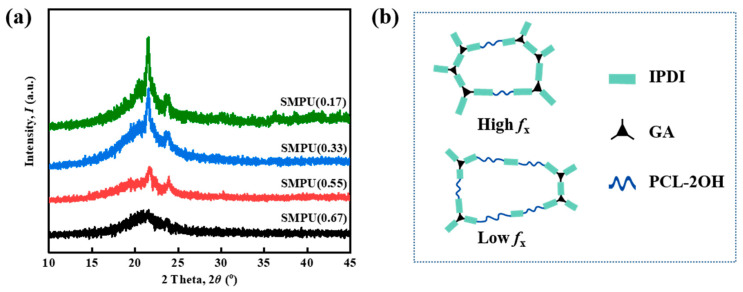
(**a**) XRD pattern and (**b**) schematic diagram of the SMPU with different crosslinking densities (*f*_x_’s).

To demonstrate the effect of crosslinking density on the crystalline properties, XRD tests are performed on the materials, and the results, as shown in Figure 10a, are consistent with the Δ*H*_m_ changes in the DSC tests. It can be seen from the figure that as the crosslinking density decreases, the crystallizability of the material gradually increases. The SMPU(0.67) with a higher crosslinking density does not show obvious diffraction peaks, but there is a steamed bread peak, which represents the formation of the amorphous phase. From SMPU(0.55) to SMPU(0.17), the diffraction peaks appear and gradually increase, which is caused by the increasing proportion of PCL-2OH in the crosslinking network. As schematically shown in Figure 10b, at lower crosslinking density, more PCL-2OH in the material reacts with IPDI to form PCL-IPDI-PCL-type connections, producing longer chain segments with stronger mobility, which is conducive to the formation of crystals. However, at higher crosslinking density, more GL reacts with IPDI in the material, more GL-IPDI-GL type connections are formed, and the movement of the PCL-2OH segment is bound, which is not conducive to the crystallization.

**Figure 11:**
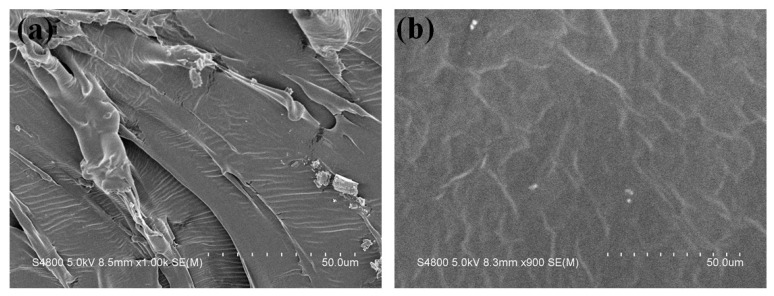
SEM images of (**a**) SMPU(0.67) and (**b**) SMPU(0.17) after brittle fracture in liquid nitrogen.

Figure 11 shows the section morphologies of SMPU(0.67) and SMPU(0.17) after brittle fracture in liquid nitrogen. It can be seen that the SMPU(0.67) exhibits ductile fracture. As the crosslinking density decreases, the material exhibits more brittle fracture. This is related to the proportion of PCL. PCL is a semi-crystalline polymer, and pure PCL shows brittleness at room temperature. In the synthesized SMPU, the crystallization performance with higher PCL content is stronger, the *T*_trans_ is higher, and it is more difficult to deform. Compared with SMPU(0.67) with higher crosslinking density, SMPU(0.17) is easier to break at low temperatures.

**Figure 12:**
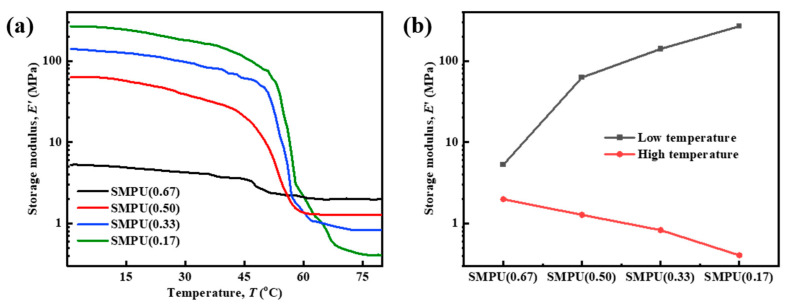
(**a**) DMTA curves showing the storage modulus during the heating of the SMPU materials with different crosslinking densities and (**b**) the comparison of the samples’ storage modulus below and above the *T*_trans_ (low and high temperatures, respectively).

The storage modulus at low temperatures reflects the anti-deformation ability of the material. The higher the storage modulus, the more difficult the material is to be deformed. The storage modulus is conducive to the shape fixation of the material. The storage modulus at high temperature reflects the mechanical properties of the material during the shape recovery, such as the size of the restoring force, and a higher storage modulus at high temperature is conducive to the shape recovery of the materials. Figure 12 shows the DMTA curves and the storage modulus comparison of SMPU as a function of temperature. The storage modulus of SMPU(0.67) at 0 °C is only 8.53 MPa, which is similar to the rubber at room temperature. There is no significant difference between the storage modulus at high temperatures and the storage modulus at low temperatures. The storage modulus of other SMPUs shows a step-like decrease around 55–65 °C with the increase in temperature, which has a numerical change of two orders of magnitude, and slightly differs from the *T*_trans_ results of the DSC tests. This is due to the fact that the bulk used in the DSC test is small, with only 5–10 mg, while the spline used in the DMA tests is larger and needs to take a certain time for the heat to transfer into the material. Moreover, the use of a frequency of 1 Hz in the DMTA tests also brings a dynamic effect. So the measured *T*_trans_ by DMTA is generally higher than the value measured by DSC, and such a difference could be as high as 15–20 °C.

**Figure 13:**
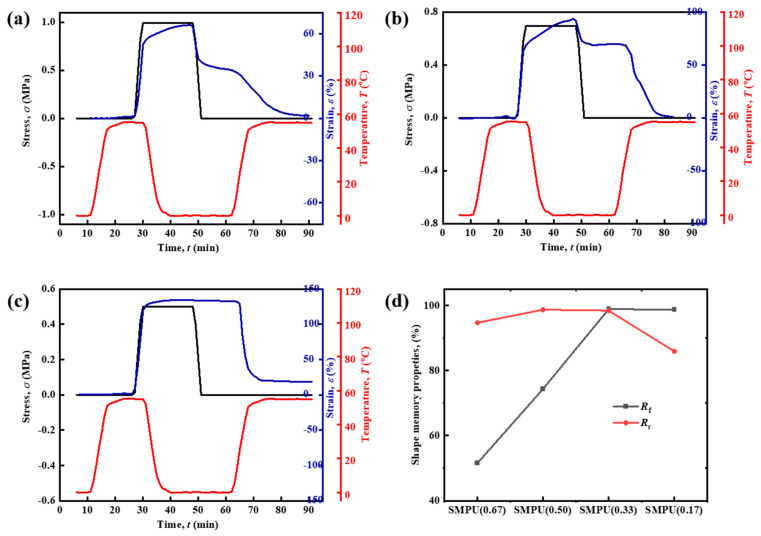
Shape memory curves of the SMPU with different crosslinking densities: (**a**) SMPU(0.67), (**b**) SMPU(0.50) and (**c**) SMPU(0.17), and (**d**) the comparison of the shape memory effect among different SMPUs.

SMPU(0.33) is exactly the SMPU-20 material discussed above, and its shape memory curve was already shown in Figure 5c. The shape memory curves of other SMPUs are shown in Figure 13. It can be seen that as the crosslinking density decreases, the stress required to strain the material increases gradually, and the *R*_f_ of the material gradually increases, reaching a shape fixation ratio of 98.8% for the SMPU(0.33), indicating that the high content of PCL proportion is good for the shape fixation. However, for the SMPU(0.17), the *R*_f_ is decreased, which may be due to the fact that the higher content of PCL reduces the crosslinking density. This creates a viscous flow strain that causes irreversible plastic deformation. It is noticed that the SMPU(0.67) does not show obvious crystallization in the XRD and DSC tests, but it shows an *R*_f_ of 51.5% in the shape memory tests. The material will continue to creep slowly if it does not start heating at the 62nd min, so its actual *R*_f_ should be lower than 51.5%.

The above results of the gel fraction, DSC, TGA, XRD, storage modulus, and shape memory performance of SMPU with different crosslinking densities show that with the decrease of crosslinking density, the proportion of PCL-2OH increases, the crystallinity of the material increases, and the shape memory performance gradually increases. However, when the crosslinking density is reduced, the shape recovery of the material is affected. After comprehensive consideration, for the SMPU(0.33) with the molar ratio of IPDI:PCL-2OH:GL of 2:1.5:0.33, the material has the best shape memory effect and comprehensive performance.

### 3.3. Shape Memory Effect Display and Mechanism Explanation

**Figure 14:**
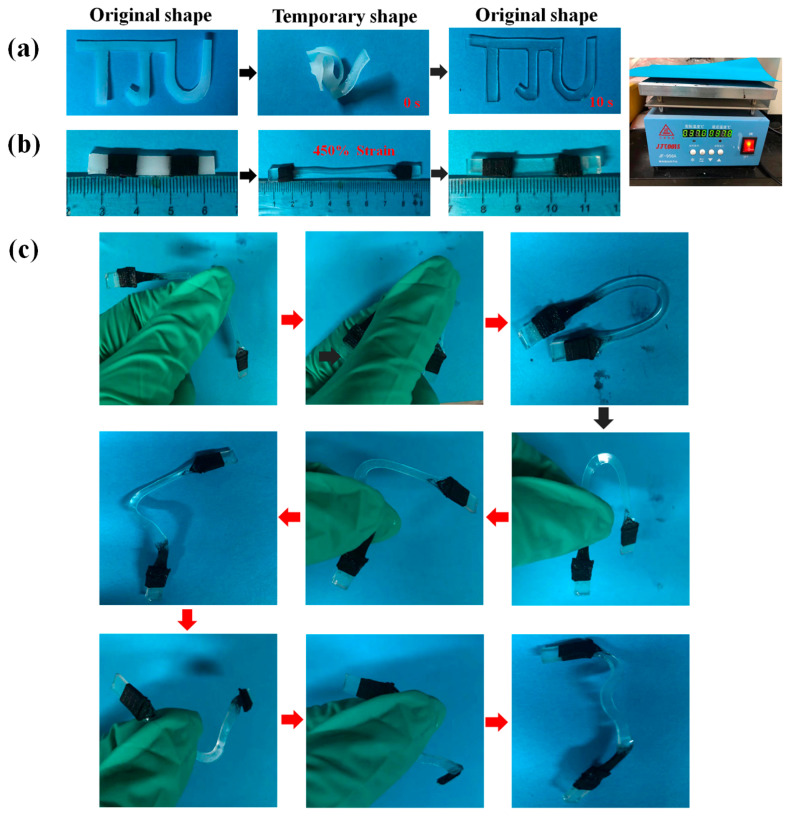
The original shape of (**a**) “TJU” shape cut out by the laser cutting machine, (**b**) 450% strained SMPU(0.33) recovered at 37 °C, and (**c**) the stretched SMPU(0.33) exhibiting complex shape changes triggered by the body temperature. The photo in the upper right corner shows the hot stage used.

Using a carbon dioxide laser cutting machine, a “TJU” shape is cut out on the SMPU film, as shown in Figure 14. As shown in Figure 14a, it is put into a water bath at 55 °C and forms an agglomerate. The temporary shape is then fixed in an ice–water mixture at 0 °C. When it is heated on a hot stage at 37 °C, the material unfolds and recovers to its original shape within 10 s. The sample is milky white at first and becomes transparent after heating to recover the original shape. This is due to the melting of crystals in SMPU and the conversion of all PCL-2OH to an amorphous phase. After standing for a period of time, part of PCL-2OH recrystallizes, and the sample will change again into a milky white opaque solid. Furthermore, as shown in Figure 14b, the SMPU(0.33) is cut into a rectangular spline of 40.0 × 5.0 × 1.0 mm^3^ and marked with black tape. It is stretched after heating, and then the temporary shape is fixed. The SMPU(0.33) can reach a huge strain of 450% and achieve nearly 100% shape recovery on the hot stage. Interestingly, as shown in Figure 14c, the stretched spline can exhibit complex shape changes triggered by the body temperature. Therefore, the prepared SMPU materials are expected to be useful in body temperature-triggered medical devices and wearable, flexible electronics.

**Figure 15:**
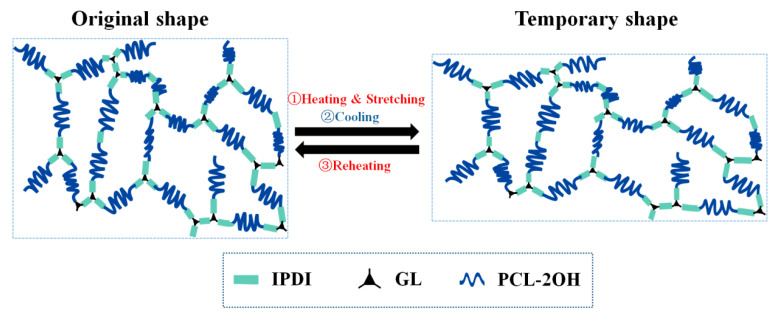
Schematic diagram of the molecular mechanism of the shape memory effect of the prepared SMPU.

Combined with the characterization results, the shape memory behavior of the prepared SMPU can be explained as schematically shown in Figure 15. There are two phases in SMPU: one is the reversible phase formed by the partial crystallization of PCL-2OH segments, and the other is the fixed phase. The material is composed of a chemically crosslinked network formed by the reaction of GL, IPDI, and part of PCL-2OH. At room temperature, part of PCL-2OH in SMPU is in a crystalline state, and the material is in a thermodynamically stable state. When heated to the *T*_trans_, the crystals melt, the movement restriction of the PCL-2OH segment is lifted, and the PCL-2OH segment is aligned and oriented along the stress direction under the external force. The macroscopic performance is that the material is stretched and elongated. In this process, the entropy of the polymer becomes lower, and the material is in a thermodynamically unstable state and has a tendency to return to the original shape. As the temperature decreases, part of the PCL-2OH segments recrystallizes, the segment movement is restricted, the temporary shape is fixed, and the elastic recovery energy is sequestered in the form of internal stress. Finally, when the SMPU is heated to the *T*_trans_ again, the PCL-2OH melts again, the SMPU returns to its original shape under the traction of internal stress, and the polymer returns to a thermodynamically stable state again.

## 4. Conclusions

In this study, a series of thermoset SMPU samples have been synthesized by a simple, time-saving, and industrialized one-step method using PCL-2OH, IPDI, and GL as raw materials. The effects of chain molecular weight and crosslinking density on the thermal properties, crystallinity, shape memory effect, and other properties of the materials have been investigated. The main conclusions can be drawn as follows:

(1) As the molecular weight of the soft chain increases, the *T*_trans_ of the materials increases gradually, the crystallinity increases, and the *R*_f_ of the material is also improved. The SMPU with a PCL-2OH soft chain molecular weight of 500 g mol^−1^ hardly has any shape memory effect, and the best shape fixation can be achieved for the molecular weights of 2000 g mol^−1^;

(2) As the crosslinking density increases, the *T*_trans_ of the materials gradually decreases, the crystallinity becomes lower, the *R*_f_ decreases, and the *R*_r_ increases. When the molar ratio of GL is 0.67, the material changes from a shape memory material to a pure elastic rubber;

(3) Using PCL220 and the molar ratio of IPDI:PCL-2OH:GL = 2:1.5:0.33, the *T*_trans_ of the materials is close to body temperature, showing the best *R*_f_ and *R*_r_, and the best comprehensive performance, which is expected to be useful in the biological medicine and other near body temperature fields.

## Figures and Tables

**Table 1:** Detailed information on PCL-2OH with various molecular weights.

Samples	Molecular Weight (g mol^−1^)	-OH Value (mg KOH g^−1^)	Viscosity (mPa s)	Melting Point (°C)
PCL205	550	204	40	18–23
PCL210	1000	112	150	30–40
PCL220	2000	56	480	40–50
PCL230	3000	37	1100	50–60

**Table 2:** Main instruments and equipment used in this work.

Instruments and Equipment	Model	Manufacturer
High-speed mixer	Dispermat AE	VMA Co., Dachau, Germany
Fourier transform infrared spectrometer	Spectrum One	Perkin-Elmer Co., Waltham, MA, USA
Scanning electron microscope	Hitachi S-4800	Hitachi Co., Tokyo, Japan
Differential scanning calorimeter	Q2000	TA Instruments Co., New Castle, DE, USA
Thermogravimetric analyzer	Q500	TA Instruments Co., New Castle, DE, USA
Dynamic mechanical, thermal analyzer	Q800	TA Instruments Co., New Castle, DE, USA
X-ray diffractometer	D/MAX-TTRIII(CBO)	Rigaku Co., Tokyo, Japan
Laser cutting machine	CT-LEG50	Beijing Chutian Laser Equipment Co. Ltd., Beijing, China
Universal testing machine	Roell BT2-FR010TE.A50	Zwick Co., Ulm, Germany

**Table 3:** *T_trans_* and Δ*H*_m_ of the PCL-2OH with different molecular weights and the SMPU.

**PCL-2OH**	**PCL205**	**PCL210**	**PCL220**	**PCL230**
*T*_m_ (°C)	36.7	42.1	45.7	51.0
Δ*H*_m_ (J·g^−1^)	76.0	78.6	75.7	73.8
Crystallinity	54.3%	56.1%	54.1%	52.7%
**SMPU**	**SMPU-05**	**SMPU-10**	**SMPU-20**	**SMPU-30**
*T*_trans_ (°C)	—	33.8	36.9	42.3
Δ*H*_m_ (J·g^−1^)	0	6.3	25.1	33.3
Crystallinity	0%	5.8%	20.6%	26.1%

**Table 4:** Detailed parameters in shape memory test of SMPU with different soft chain molecular weights.

Samples	*ε*_load_ (%)	*ε*_fix_ (%)	*ε*_rec_ (%)	*R*_f_ (%)	*R*_r_ (%)
SMPU-05	82.4	3.1	0.6	3.8	82.1
SMPU-10	120.8	92.0	1.7	76.1	98.2
SMPU-20	97.1	96.0	1.6	98.8	98.4
SMPU-30	120.6	119.2	1.9	98.9	98.7

**Table 5:** The *T*_trans_ and Δ*H*_m_ of the SMPU with different crosslinking densities.

Samples	SMPU(0.67)	SMPU(0.50)	SMPU(0.33)	SMPU(0.17)
*T*_trans_ (°C)	32.3	34.2	36.9	39.7
Δ*H*_m_ (J·g^−1^)	-	2.19	25.1	30.7

## Data Availability

Most of the datasets supporting the conclusions of this article are included within this article. The datasets used or analyzed during the current study can be shared with permission from the corresponding author upon reasonable request.
